# Fabrication and Photoelectrochemical Activity of In_2_S_3_ Infused TiO_2_ Nanorod Heterostructure Photoelectrodes for Solar Water Splitting

**DOI:** 10.3390/nano16010044

**Published:** 2025-12-29

**Authors:** Aravindha Raja Selvaraj, Kasinathan Kasirajan, Jaehyun Hur

**Affiliations:** 1 School of Chemical, Biological and Battery Engineering, Gachon University, Seongnam-si 13120, Gyeonggi-do, Republic of Korea; rajanano12@gmail.com; 2 Division of Advanced Materials Engineering, Kongju National University, Budaedong 275, Seobuk-gu, Cheonan-si 31080, Chungnam, Republic of Korea

**Keywords:** photo anodes, photoelectrochemical, water splitting, semiconductor, heterostructure

## Abstract

Titanium dioxide (TiO_2_) and indium sulfide (In_2_S_3_) combined nanoarray films, fabricated via the hydrothermal and chemical bath deposition (CBD) methods, were employed as photoelectrocatalysts for water splitting applications through the photoelectrochemical (PEC) process. The resulting heterostructure nanoarray catalyst morphology, composition, and optical absorption have been analyzed. The photon illumination and its effect on the electrochemical impedance and photocurrent generation measurements exposed that the infusion of In_2_S_3_ on the TiO_2_ films comprehensibly reduced the charge carrier transport resistance (700 Ohm·cm^2^) and enhanced the photocurrent (0.28 mA/cm^2^) with an increment of photo potential response (−1.02 V vs. Ag/AgCl). Further, the heterostructure films effectively degrade the organic molecules in the electrolyte under UV light illumination. The enhanced catalytic reaction is ascribed to the role of In_2_S_3_ deposition on the TiO_2_, which effectively improves the charge carrier collection at the surface by In_2_S_3_ and promotes the dissociation of organic molecules at the interface.

## 1. Introduction

Electrochemical hydrogen evolution as a renewable source is the appropriate method to obtain green energy gas due to high gravimetric energy density (120 MJ/kg) [1]. Compared with other methods, the photo(electro)catalytic method provides water splitting into hydrogen in an unpretentious technique to meet urgent energy and environmental requirements [1,2,3]. However, deficient surface reaction sites and photogenerated electron–hole pairs from the semiconductor generate a lower volume of hydrogen [4,5]. The surface and electronic states of semiconductor photoelectrocatalyst development need to play pivotal roles in addressing these challenges.

Recently, heterostructure photo electrocatalysts have provided great advantages in photon-irradiated electron–hole pair separation and conduction due to the in-built electric field development under illumination with favorable band edges [6,7,8]. Under photoirradiation, the photoexcited electron and hole carriers transport confirm light-induced carriers in the semiconductors that enable photocatalytic redox reaction for hydrogen evolution [9,10,11]. In a single material semiconductor interface, the surface recombination occurrence is higher during the redox reaction [12,13,14,15]. For this, a staggered heterostructure photocatalyst is used to increase the successful transfer of the carriers [16,17]. The carrier’s dynamics of the photo absorptive materials at the top of the layer with a suitable band edge favor the hydrogen evolution reaction [18]. Hence, integrating a heterostructure photo electrocatalyst with a wideband gap base layer as the electron collector could effectively induce the carrier’s recombination [19]. Likewise, the photoexcited holes from the oxidative layer rapidly transfer to the electrochemical cell for the water splitting reaction [20]. The separation of photo-irradiated electrons and holes strongly develops the internal built-in field in the photoanode [21].

Metal sulfides are considered promising photocatalysts for water splitting applications owing to designable heterostructures, suitable energy band gaps, and superior photoelectric characteristics [22,23,24]. Among the diverse metal sulfide materials, indium sulfide (In_2_S_3_) stands out as a highly promising, chemically stable, and low-toxicity rare-earth molecular compound for water splitting applications [25,26]. Its band gap energy, typically ranging from 1.9 to 2.4 eV, is particularly suitable as it enhances visible light absorption [27,28,29,30]. The micromorphological In_2_S_3_ and its heterostructures with TiO_2_, ZnO, WO_3_, In_2_O_3_, Fe_2_O_3_, CuO, and NiO metal oxides effectively increase light-induced photocurrent densities from 0.1 µA/cm^2^ to 1 mA/cm^2^ [31]. The dimensionally modulated nanostructure of In_2_S_3_ effectively increases the catalytic reaction in the water oxidation reaction [32]. To enhance catalytic efficiency by improving the surface area, various nanostructures, including nanorods, nanospheres, and nanosheet morphologies have been achieved using diverse wet-chemical and energy-assisted synthetic techniques, such as microwave irradiation, sonochemical, and hydrothermal/solvothermal methods [33,34,35]. Also, the combination of metal oxide with In_2_S_3_ has been widely studied as a photoelectrocatalyst for renewable [29] and optoelectronic applications [30], including photoanode devices [36]. The metal oxide layers with 1D nano-surface morphology, grown by hydrothermal and CVD techniques, are used to improve the surface area of In_2_S_3_ [37,38,39]. The metal oxide layer holds good stability and forms heterojunctions with In_2_S_3_ [40].

To reduce recombination, the TiO_2_ nanoarrays passivated with various nanoparticles with metal sulfide heterostructures are a recent, facile technique inherited by modern researchers [41,42]. The In_2_S_3_ nanoparticles on TiO_2_ nanorods enhance the photoinduced charge carrier separation to 0.2 mAcm^−2^ [43]. In addition, the metal sulfides’ heterostructure has been made to absorb more photons in the visible range than TiO_2_, which absorbs mostly UV photons. The narrow band gap metal sulfides (CdS, CdSe, CuS, ZnS) strongly make a heterostructure with TiO_2_ and improve the inbuilt potential based on the band edge, which decides the reaction type in the heterostructure materials. However, the heterostructure of TiO_2_ with CdS, ZnSe, CdSe, PbS, ZnS reported a reasonably good performance and exhibited good electrochemical properties [44,45,46,47,48]. The photo corrosion effect is inevitable in the metal sulfide photo catalysts; however, In_2_S_3_ strongly holds the stability against photo-corrosion due to the ternary sulfide phase and charge carrier flow by a type II heterostructure [34,35].

In the present work, we examined the TiO_2_ and In_2_S_3_ heterostructure photoelectrode for PEC water splitting applications. The nanoarray morphology and passivation of In_2_S_3_ particles around TiO_2_ nanorods have been established by a two-step synthesis method. The photoelectrocatalytic water-splitting capabilities of TiO_2_/In_2_S_3_ have been examined. The photocurrent densities, charge carrier transport mechanisms, environment remediation, solar energy to hydrogen conversion, and applied bias-based photon-to-electron conversion have been analyzed. The resulting TiO_2_/In_2_S_3_ nanoarrays exhibit a convenient, simple fabrication process and are highly efficient for water splitting abilities and hydrogen evolution applications.

## 2. Experimental Methods

### 2.1. Materials

The chemicals are analytical reagent grades, which have been used without further processing. The titanium isopropoxide (TIP, 98%, Daejung, Deajeon, Republic of Korea), concentrated HCl (35.46%, Daejung, Daejeon, South korea extra pure), InCl_3_ (98%, Sigma-Aldrich, St. Louis, MO, USA), citric acid, and thioacetamide (Daejung, extra pure, Daejeon, Republic of Korea). The chemical solutions were prepared using deionized water. The F: SnO_2_ deposited glass slides were used as a substrate. Initially, the substrates were ultrasonically cleaned according to the standard cleaning procedure. The F:SnO_2_ deposited glass slides (~7 Ω/cm, 1 cm × 1.5 cm × 2 mm) were used as substrates for all photoelectrochemical measurements and dye degradation experiments.

### 2.2. Preparation of TiO_2_ Photoanodes

**Figure 1:**
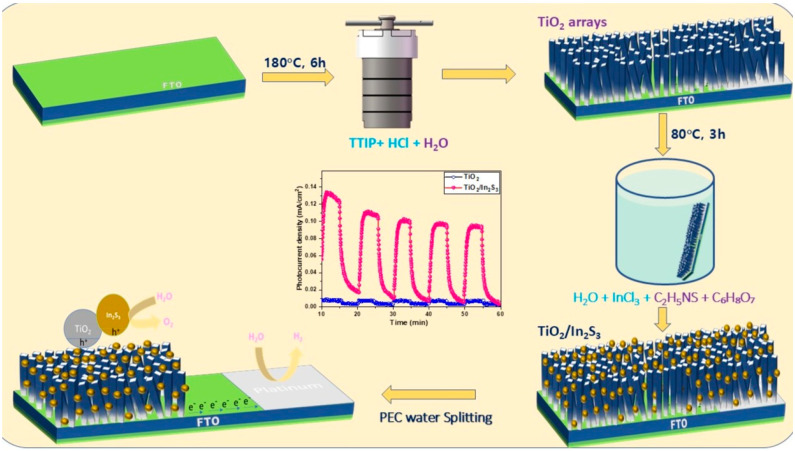
Schematic diagram of growth and PEC water splitting of TiO_2_/In_2_S_3_ photoanode.

In the growth experiment (Figure 1), the precursor solution was formulated by combining titanium alkoxides in aqueous media with warm water, utilizing concentrated HCl as a hydrolysis catalyst for hydrothermal growth. The hydrochloric acid was dropped into the solution as a catalyst, and the pH of the solution was adjusted to a strong acidic nature. Hydrochloric acid plays a role in the hydrolysis (Equations (1) and (2)) of the precursor and lowers the surface kinetic energy of the TiO_2_ growth plane and side wall, promoting the one-dimensional growth on the FTO. The hydrothermal reaction is as follows at 180 °C for 6 h.

**Ti(OR)_4_ + 4H_2_O → Ti(OH)_4_ + 4ROH** (1)

**Ti (OH)_4_ → TiO_2_+ 2H_2_O** (2)

### 2.3. Preparation of In_2_S_3_ Infused TiO_2_ Photoanodes

In_2_S_3_ nanoparticles were deposited on the TiO_2_ nanorods using a simple chemical bath deposition (CBD) process. In a typical growth process, 0.01 M of indium chloride, 240 mg of thioacetamide, and 480 mg of citric acid monohydrate were dissolved in 20 mL of water. The dissolved solutions have been added and stirred vigorously for 15 min at room temperature. The resultant clear solution was then transferred to a constant temperature bath at 80 °C. The synthesized TiO_2_ nanorods have been immersed in the solution to deposit the In_2_S_3_ on the nanorods. The reaction has been carried out for 3 h and cooled naturally to room temperature. The deposited orange-yellow colored In_2_S_3_ photoanode was washed several times with ethanol and dipped in deionized water to eliminate the unreacted constituents from the nanorod surface of the photoanode and annealed at 150 °C for 3 h.

### 2.4. Preparation and Characterization of Photoanodes

The surface view of TiO_2_ and In_2_S_3_-infused TiO_2_ was examined by field emission scanning electron microscopy (FESEM, Carl Zeiss and Oxford instruments, Gyeongsan. The crystalline structures of the prepared photoanode were identified using an X-ray diffractometer (XRD, Pan Analytical Pro, Gyeongsan, Republic of Korea) with Cu Kα radiation (λ = 0.15406 nm), and the acceleration voltage and the applied current were 40 kV and 40 mA, respectively. X-ray photoelectron spectroscopy (XPS, Gyeongsan) was performed on a K-alpha, Thermo Scientific, USA, using Al K_α_ as the X-ray source (1486.6 eV). Photoluminescence (PL) spectra were obtained by a Horiba spectrometer-Japan, (Gyeongsan) with an excitation source of a He-Cd laser (325 nm).

### 2.5. Photoelectrochemical Measurements

Photoelectrochemical analyses were performed using a 0.1 M Na_2_SO_3_ electrolyte solution in the Corr test electrochemical workstation. The three-electrode system of TiO_2_ and In_2_S_3_/TiO_2_ photoanode as the working electrode, Pt plate as the counter electrode, and an Ag/AgCl as the reference electrode was used. A xenon lamp (Oriel LCS, Republic of Korea) is used for a solar light source with a UV cut-off filter. Electrochemical impedance spectra (EIS) were recorded at a 0.1 V potential in the 10 Hz to 10 K Hz band frequencies. The photocurrent output was recorded for the bias potential of −0.95 to 0.2 V with a sweeping voltage of 20 mV.

### 2.6. PEC Degradation Experiments

The dye degradation experiments were tested using a photoanode. In an unbiased condition, the working electrode and electrolyte solution with organic dye molecules have been maintained in a neutral condition (pH = 7). The PEC reactions of the photoactive working electrode were assessed by the dissociation of rhodamine B concentration. In a characteristic process, the photoelectrode was kept in an apparatus comprising the dye solution, ensuring dye adsorption/desorption by the photoelectrode. At intervals of time, the degraded dye samplings have been visibly ensured.

## 3. Results and Discussion

### 3.1. UV–Visible Light Absorption

**Figure 2:**
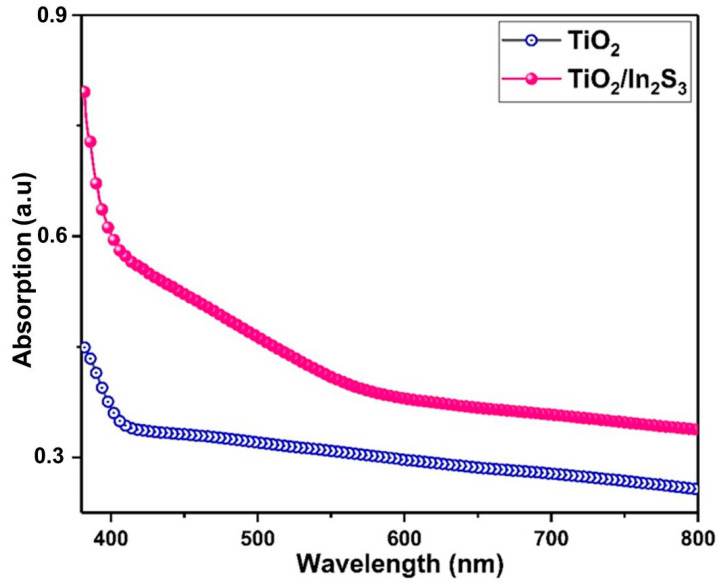
UV–visible light absorption spectrum of the TiO_2_ and TiO_2_/In_2_S_3_.

Figure 2 shows the results of analyzing the TiO_2_ and TiO_2_/In_2_S_3_ photoanodes’ UV–vis absorption spectra, which reveal their visible light absorption characteristics. The TiO_2_ photoanode displays stronger UV light absorption, and In_2_S_3_-infused TiO_2_ increases the light absorption capabilities more than TiO_2_. After In_2_S_3_ is infused in TiO_2_, the heterostructure photoanode exhibits better optical properties with an extended reduction in the reflectivity, and the absorption edge increases to 500 nm. The vertical TiO_2_ nanorods allow the incident light to reflect several times between the nanorods clearly explained the advanteges of nanorods structure. More photons are absorbed by the semiconductor and produce more photo excitons in the photoanode.

### 3.2. Morphology Characterization

**Figure 3:**
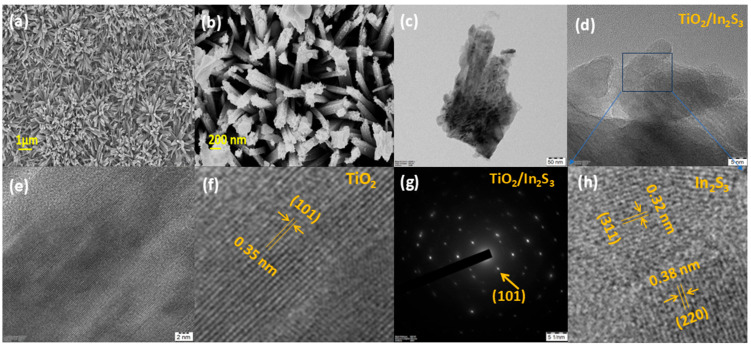
FESEM images of (**a**) TiO_2_ and (**b**) TiO_2_/In_2_S_3_ nano-arrays. (**c**) TEM images of the TiO_2_/In_2_S_3_. (**d**) HRTEM images of the TiO_2_/In_2_S_3._ (**e**) The fringe pattern of TiO_2_. (**e**) The low magnification of TiO_2_ nano rods. (**f**) The fringe pattern of (101) plane of TiO_2_ nanorods. (**g**) The selective area electron diffraction pattern of the TiO_2_/In_2_S_3_. (**h**) The magnified view of the fringe pattern of In_2_S_3_ in TiO_2_/In_2_S_3_ heterostructure.

To analyze the surface texture, field emission scanning electron microscope (FESEM) analyses were performed for the TiO_2_, TiO_2_/In_2_S_3_ (Figure 3). From Figure 3a, it is assessed that the TiO_2_ surface morphology has a nanorod shape structure, which is consistent with the previous reports [49,50]. The typical nanorod image (Figure 3a) states that the diameter of the rods is approximately 150 nm and the length of the nanorod is approximately 1 µm, which is evidence that the growth of TiO_2_ has a large surface area. The nanorod morphology of the TiO_2_ substantially interacts with the dye molecules as physisorption and admits to dissociation through photo-eletro catalytic reactions [51]. In contrast, with the addition of In_2_S_3,_ more void structures have been introduced at the surface (Figure 3b). TiO_2_ nanorods expose top and sidewall facets clearly. A surface-view SEM image (Figure 3b) reveals the actual surface morphology of pure TiO_2_ nanorod array films. From Figure 3b, the In_2_S_3_ particles are spread on the TiO_2_ nanorods uniformly and cover the entire TiO_2_ surface.

The high magnification HRTEM images of TiO_2_/In_2_S_3_ nanoarray reveal that the interface of the semiconductors is a heterostructure formation (Figure 3c–h). The higher magnification, shown in Figure 3f, reveals well-defined and clear lattice fringes for the {101} planes of TiO_2_ nanorods with a d-spacing of 0.35 nm, which is in excellent agreement with the tetragonal anatase phase of TiO_2_ [45,46]. Similarly, from the HRTEM image(Figure 3h), the In_2_S_3_ particle on the TiO_2_ nanorods exhibits the fringe pattern for the {311} and {220} planes with the d-spacing of 0.32 and 0.38 nm, respectively. The SAED pattern (Figure 3g) for the In_2_S_3_/TiO_2_ heterostructure clearly indicates the crystalline phase of the TiO_2_ for the (101) plane and the low crystalline phase for the In_2_S_3_ nanocrystals. The distinct observation of characteristic lattice fringes for both TiO_2_ and In_2_S_3_ within the morphological region provides direct crystallographic evidence for the formation of a heterostructure, facilitating the efficient charge transfer and enhanced photoelectrochemical performance. The elemental composition and ratio of the TiO_2_/In_2_S_3_ nanoarray are confirmed by Energy-Dispersive X-ray Spectroscopy (EDS) spectrum (Figure S1), clearly showing the presence of all elements, and the calculated atomic weight percentages from EDS were Ti (55.30%), O (37.25%), In (6.20%), and S (1.25%). The quantifiable detection of indium and sulfur confirms the synthesis and integration of the In_2_S_3_ layer in the TiO_2_ nanoarrays.

### 3.3. XRD Analysis

**Figure 4:**
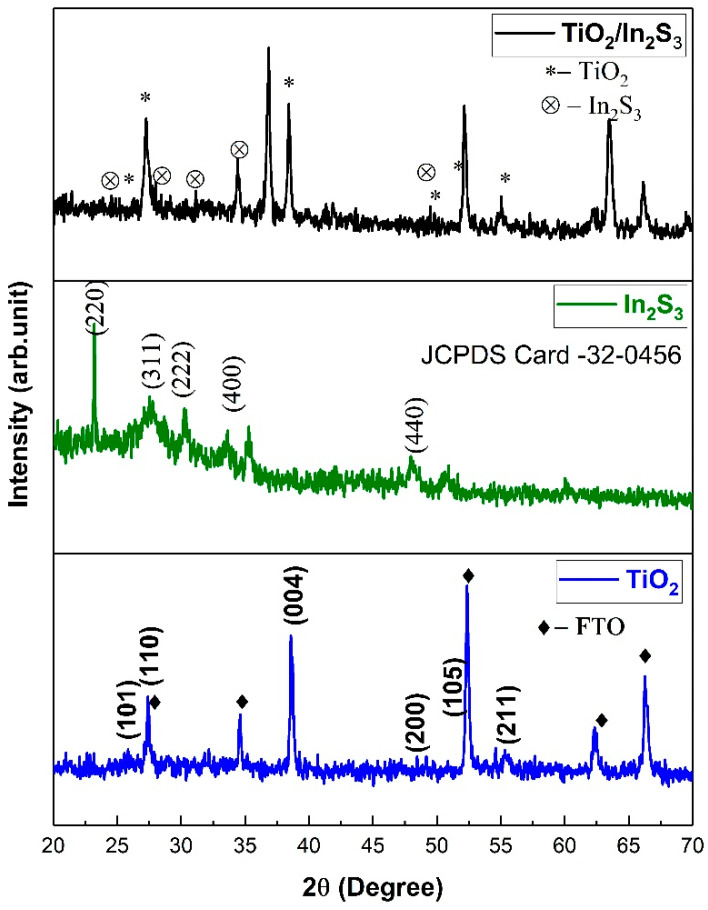
The XRD pattern of TiO_2_, In_2_S_3_ and TiO_2_/In_2_S_3_.

Figure 4 illustrates the XRD pattern corresponding to the as-synthesized TiO_2_ nanorods, In_2_S_3,_ and TiO_2_/In_2_S_3_. The heterostructure thin films were confirmed by their respective crystallographic planes. The diffraction peak of TiO_2_ nanorod array film comprises the mixed anatase and rutile phases of TiO_2_. The presence of diffraction peaks at 25.31°, 37.83°, 48.02°, 53.95°, and 55.12° is assigned to the (101), (004), (200), (105), and (211) crystal planes of the anatase phase of TiO_2_ (JCPDS No. 21-1272). The typical diffraction peaks at 27.42° and 36.12° reveal the (110) and (101) planes in the rutile phase of TiO_2_ [52]. The strong peaks at 26.53, 33.64, 51.51, 61.72, and 65.52 are ascribed to the FTO-coated glass substrates [53], and no diffraction peaks related to the In_2_S_3_ were observed superficially due to the low concentration of In_2_S_3_ (0.1 mg), which has been confirmed further by the SAED for the heterostructure films. However, the XRD pattern of the In_2_S_3_ thin film reveals the diffraction peaks at 23.40°, 27.34°, 32.68°, 33.24°, and 47.72°, which can be indexed to miller indices at (220), (311), (222), (400), (331), and (440) of the JCPDS (00–032–0456), and confirm strong In_2_S_3_ formation over the chemical bath deposition synthesis [54]. Also, from the diffraction pattern of the In_2_S_3_/TiO_2_ film having the asymmetric peak for the (110) plane, which shows strong metal–oxygen influence, the presence of the O-In-S system could be infered [55,56].

### 3.4. XPS Analysis

**Figure 5:**
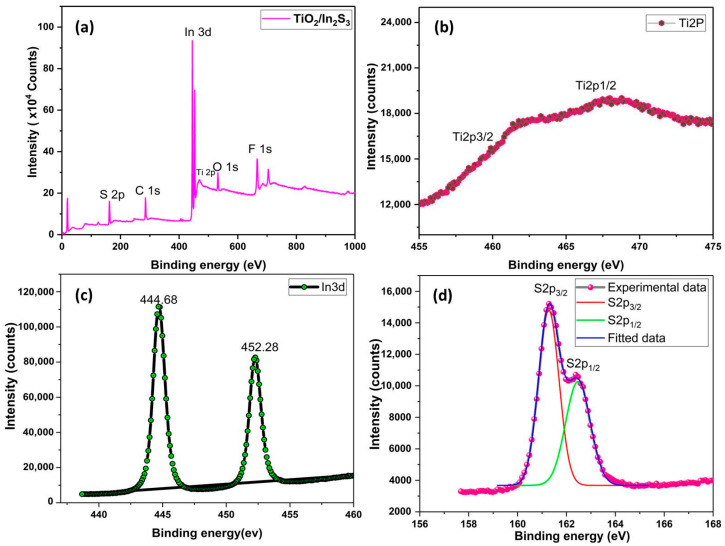
(**a**) XPS survey scan, (**b**) Ti 2p, (**c**) In 3d, and (**d**) S 2p of TiO_2_/In_2_S_3_ hetero structure photoanode.

The surface elemental composition of the TiO_2_ nanorods and In_2_S_3_ heterostructure film is studied by the high-resolution XPS spectra that were collected, which are depicted in Figure 5. In Figure 5a, the survey spectra for TiO_2_/In_2_S_3_ nanoarray thin films are shown. The TiO_2_ layer is confirmed by the binding energies of titanium (Ti 2p) and oxygen (O 1s), while the In_2_S_3_ heterostructure is formed by sulphur (S 2p) and indium (In 3d). The presence of Sn and F peaks, expected from the fluorine-doped tin oxide (F:SnO_2_) substrate, is also confirmed. The XPS peak of C 1s at 285.08 eV is assigned to the carbon residual. From the high-resolution Ti 2p spectrum (Figure 5b), peaks located at 459.58 eV and 465.8 eV are observed to be Ti 2p_3/2_ and Ti 2p_1/2_ orbitals of Ti^4+^ [35]. The higher binding energy values of the Ti 2p intimate the tight binding of the heterostructure of TiO_2_/In_2_S_3_. Figure 5c shows the XPS spectrum of In 3d with two symmetrical peaks at the binding energies of 444.4 eV for In 3d_5/2_ and 452.28 eV for In 3d_3/2_ [35,57]. The S 2p peaks (Figure 5d) at 162.82 and 161.13 eV were defined to S 2p_1/2_ and S 2p_3/2_, respectively, which clearly indicates the absence of S-O bonding in the In_2_S_3_ phase [58,59]. From Figure S2, the O1s peak is assigned at 530.21 to 531.9 eV, which agrees with (Ti–O) lattice oxygen and hydroxyl groups (–OH) [59,60]. From the analysis of XRD, SEM, and XPS, the existence of In_2_S_3_/TiO_2_ heterostructures was confirmed.

### 3.5. PL Spectra Analysis

**Figure 6:**
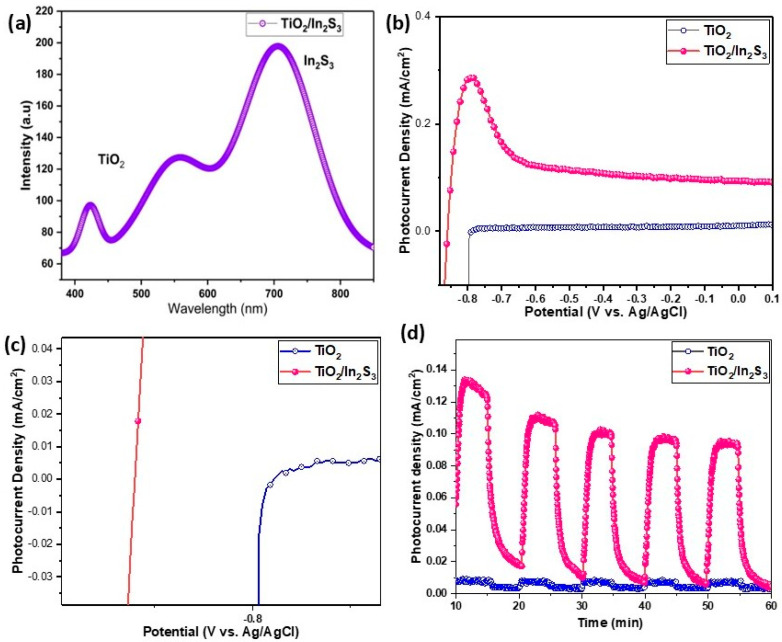
(**a**) The photoluminescence spectrum of the TiO_2_/In_2_S_3_ photoanode, (**b**) LSV graph of TiO_2_ and TiO_2_/In_2_S_3_ photoelectrodes, (**c**) onset potential negative shift for TiO_2_/In_2_S_3_, and (**d**) chronoamperometric photocurrent stability of photoelectrodes.

The photo-induced carriers in excitation and recombination have been measured using photoluminescence spectra (Figure 6a). The electron–hole pairs are produced when the TiO_2_ nano-array is excited by near-UV light. The photoexcited electrons from the conduction band of TiO_2_ will experience a shift to the valence band, ensuring a low emission intensity [61]. Figure 6a shows the PL spectra of the TiO_2_/In_2_S_3_ heterostructure film. Visibly, In_2_S_3_ displays a substantial effect on the PL intensity, which shows that a greater number of photoexcitations occurred in In_2_S_3_. In the case of the In_2_S_3_/TiO_2_ heterostructure, photoexcited carriers of In_2_S_3_ were injected into TiO_2_ and inhibited the recombination in the In_2_S_3_ surface layers. The PL band edge results show a feasible path under photoexcitation. The parting of electron–hole pairs to the redox reaction is enabled by a high number of photoexcited holes compared to the TiO_2_, which enhances the hydrogen evolution for the heterostructure photoanodes (TiO_2_/In_2_S_3_).

### 3.6. Photoelectrochemical Performances of Photoelectrodes

#### 3.6.1. Photocurrent Measurements

The photocurrent evolution to the respective applied bias potential and time has been examined as linear sweep voltammetry and chronoamperometry analysis (Figure 6b,d). Noticeably, under solar light illumination, the photocurrent of the TiO_2_ photoelectrodes increased progressively and remained stable up to 0.1 V vs. Ag/AgCl with the increase in bias potential. At the wide potential range (−0.9 to 0.1 V vs. Ag/AgCl), the TiO_2_ photoanode shows stable photocurrent density, which ensures the stability and redox reaction of the photoelectrodes. However, the TiO_2_ photoanode exhibited a comparatively low photocurrent compared to TiO_2_/In_2_S_3_, which indicates that more photoexcited carriers developed in the TiO_2_/In_2_S_3_ electrodes. Precisely for the TiO_2_ electrode, it generates 0.5 µA·cm^−2^ photocurrent at −0.6 V vs. Ag/AgCl. After infusing In_2_S_3_ into TiO_2_ nanoarrays, the photocurrent of TiO_2_/In_2_S_3_ rises distinctly to 0.28 mA·cm^−2^ at −0.8 V vs. Ag/AgCl. The onset potential has been found as −0.8 V vs. Ag/AgCl for the TiO_2_, and it has been improved with the infusion of In_2_S_3_ as −0.9 V vs. Ag/AgCl. The instant response to the illumination has been identified by the transient linear sweep measurement. The very low dark current shows that there is no ionic conduction process between the electrodes. The photocurrent density as a function of time measurement (Figure 6d) shows a 95% photocurrent retention over the 1 h period for In_2_S_3_/TiO_2_ photoelectrodes. The outcome is very consistent with the photoluminescence spectra and impedance results, which ensures the enhancement of the photoactivity of the electrodes.

#### 3.6.2. EIS Measurements

**Figure 7:**
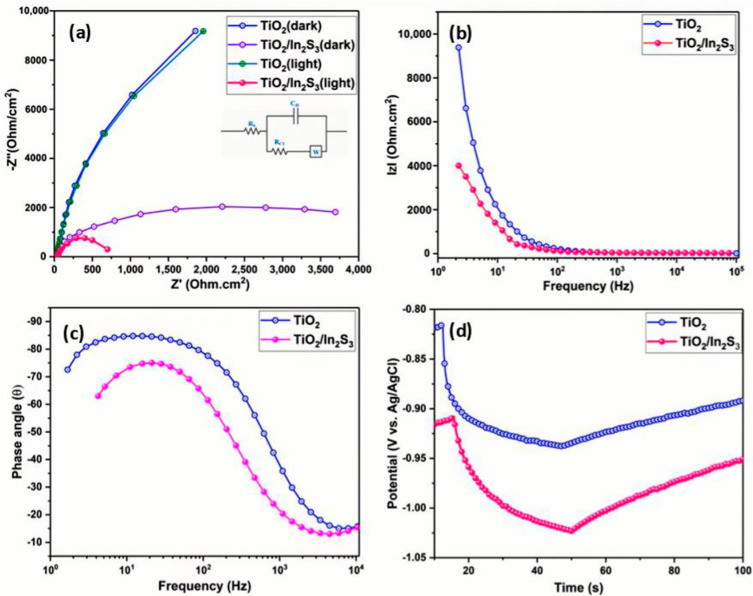
(**a**): Electrochemical impedance spectrum of TiO_2_, TiO_2_/In_2_S_3_ photoelectrodes, (inset: equivalent circuit diagram of EIS), (**b**) Bode de magnitude plot for the TiO_2_, TiO_2_/In_2_S_3_ photoelectrodes, (**c**) Bode e magnitude plot for the TiO_2_, TiO_2_/In_2_S_3_ photoelectrodes, and (**d**) open circuit potential of TiO_2_, TiO_2_/In_2_S_3_ photoelectrodes.

Figure 7a shows the EIS response of the TiO_2_ and TiO_2_/In_2_S_3_ under visible light illumination. The radius of the Nyquist plot shows the carrier transfer resistance during the reaction. The circuit resistance of the TiO_2_ (27 Ohm·cm^2^) photoanode is increased to 35 Ohm/cm^−2^ (TiO_2_/In_2_S_3_). In the absence of visible light illumination, the TiO_2_ photoanode exhibits a resistance of 8000 Ohm.cm^2^ at 0.4 V vs. Ag/AgCl. Under light illumination, an inbuilt potential is developed in the photoanode, facilitating the movement of charge carriers to the reaction site. The In_2_S_3_ passivated TiO_2_ exhibits 750 Ohm·cm^2^. The photoanode generally exhibits a CPE value on the order of 3.16 × 10^−5^ F, which is used as a fitting parameter to conclude the charge transport resistance. The in situ grown In_2_S_3_ on TiO_2_ nanorods with a nanostructured surface and vertical nanorod structure accelerates the photoexcited carriers towards the FTO film. The crystalline TiO_2_ has a continual and well-ordered interior structure, which makes a high electric conductivity under photoexcitation and an inbuilt potential established by the heterostructure [62,63]. Furthermore, the bare facets of TiO_2_ and In_2_S_3_ have participated in the redox reaction by transferring carriers to the electrolyte for hydrogen evolution. The low resistance for the In_2_S_3_/TiO_2_ reveals a tolerance of more photoinduced holes at the interface and involves the redox reaction. As reported previously, the reaction rate increasing at the surface can be validated as higher conductivity and low resistance behavior of the photoanodes [64,65,66]. The lowest real part of the impedance at higher frequencies substantiates the faster redox reaction by the photoelectrode. The results reveal the In_2_S_3_/TiO_2_ photoanodes ability to hold the highest PEC efficiency.

#### 3.6.3. OCP and Charge Transport Properties

**Figure 8:**
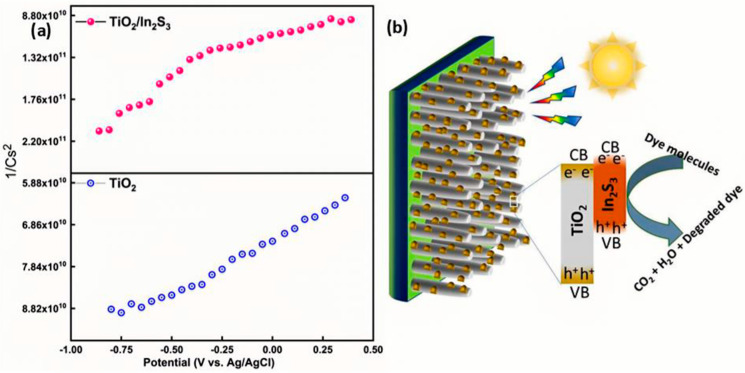
(**a**): Mott–Schottky plot for the TiO_2_, TiO_2_/In_2_S_3_ photoelectrodes, and (**b**) schematic photo (electro) catalytic redox reactions on the photoelectrodes. The more photoexcitated In_2_S_3_ molecule under visible light degrades the dye molecule into the small fragments of organic moieties.

For a complete understanding of the photoexcited charge carrier process at the interface of electrode and electrolyte, the PEC impedance spectra have been measured and shown in Figure 7b. TiO_2_ exhibits high resistance to moving the charge carriers for the redox reaction. However, the In_2_S_3_ deposition on the TiO_2_ visualizes low resistance, which indicates that a greater number of photoexcited carrier transfers have happened in a certain time. The enhanced photoexcited hole transfer leads to an enhanced photocurrent. However, the Bode phase plots in Figure 7c,d provide perceptions of the carrier kinetics in the photoelectrode. The photoelectrodes’ characteristic relaxation frequency peaks at 12 and 28.1 Hz for TiO_2_, and TiO_2_/In_2_S_3_ implies a decrease in electrochemical process time for the heterostructure electrode. In addition, the peak shift means that the photoelectrode progressively accelerates the redox reaction at the interface and significantly reduces the phase value. The low surface recombination indirectly means that a high number of carriers are present in the photoelectrode for the redox reactions, which is further evidenced by an open-circuit potential (OCP) measurement, as shown in Figure 7d. The built-in potential development by the photoelectrode demonstrates the OCP value from −0.93 V vs. Ag/AgCl to −1.02 V vs. Ag/AgCl, under light illumination. The negative deployment of the OCP validates the n-type semi-conductance of the photoelectrode. The heterostructure displays a larger photovoltage (*V*_ph_ = OCP_dark_ − OCP_light_), indicative of 0.122 V vs. Ag/AgCl for TiO_2_ and 0.126 V vs. Ag/AgCl for In_2_S_3_/TiO_2_, which shows that the In_2_S_3_/TiO_2_ photoanode has suppressed surface carrier recombination. The slow decay of the OCP indicates longer lifetime spans of carriers at the interface of the TiO_2_/In_2_S_3_, which has been confirmed with the growth and decay time of the photoanodes (Figures S3 and S4). The Bode magnitude plot reveals that the conductance of the carriers is higher at TiO_2_/In_2_S_3_. The Mott–Schottky analysis (Figure 8a,b) indicates that the shift of flat band potential towards more negative (0.99 V vs. Ag/AgCl) values for the TiO_2_/In_2_S_3_. The TiO_2_ photoanode has a flat band potential of 0.64 V vs. Ag/AgCl. The carrier concentration and space charge layer have been calculated using the Mott–Schottky relations [67]. The calculated amount of increased n-type carriers (6.13 × 10^17^ cm^−3^) with the decrease in space charge layer width (W_SCL_) of 10 nm is the reason for localizing the higher collection efficiency of charges in the TiO_2_/In_2_S_3_ photoanode. Moreover, the flat band potential difference of 0.35 V vs. Ag/AgCl between the TiO_2_ and TiO_2_/In_2_S_3_ confirms the type II conduction band edges between the semiconductors.

## 4. Photocatalytic Reaction Mechanism

A thorough investigation of H_2_ production features has been conducted by the fabrication of the photoanode and counter electrode integration. Figure 8b shows the wireless cell configuration for the light-induced hydrogen generation from the photoanodes of TiO_2_/In_2_S_3_ and Pt electrode components. The In_2_S_3_ acts as a visible light absorber, and the photoexcited charges have been transported to the FTO through the higher band gap of TiO_2_ nanorods. The interface between the TiO_2_ and In_2_S_3_ stores the electron for a certain time and allows the redox reaction to acquire the hydrogen from the counter electrode. The photoelectrochemical studies ensure that the long lifetime of the charge carrier is converted into hydrogen through the water splitting reaction. The In_2_S_3_/TiO_2_ photoanodes work under visible light, as well as UV light irradiation. To confirm the redox reaction, an organic molecule has been tested for a one-hour duration (Figure 8b). Utilizing the redox reaction, the TiO_2_/In_2_S_3_ photoanode has been used for hydrogen generation activity in environmental remediation applications.

### 4.1. Applied Bias Photon-to-Current Efficiency (ABPE)

**Figure 9:**
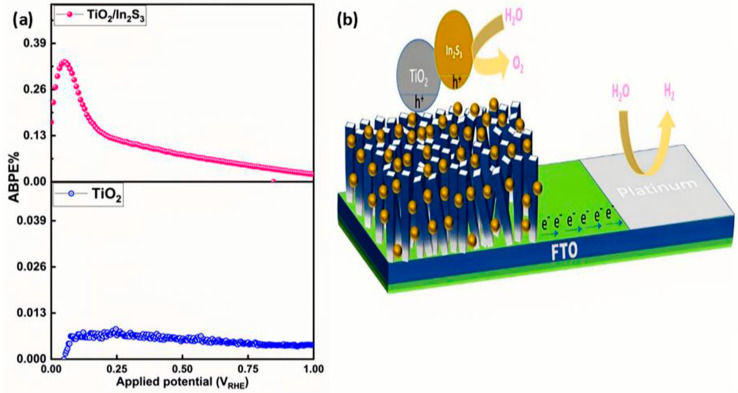
(**a**) ABPE conversion of TiO_2_, TiO_2_/In_2_S_3_ photoelectrodes, and (**b**) the schematic view of the redox reaction mechanism in the TiO_2_/In_2_S_3_ photoanode.

Applied bias photon-to-current efficiency (ABPE) has been measured as an important index to evaluate the hydrogen generation in photoelectrochemical water splitting, which is directly reflected in the half-cell solar-to-hydrogen (HSTH) conversion efficiency. The ABPE values have been calculated from the LSV measurement. The ABPE value of the TiO_2_ nanorods array was only 0.007% at 0.1 V (vs. RHE), while the ABPE values of the TiO_2_/In_2_S_3_ photoanode amplified to 0.33% at 0.04 V (vs. RHE) maximum ABPE value (Figure 9a).

### 4.2. Solar to Hydrogen Generation

Here, a plausible technique for photocatalytic, as well as photo-electrocatalytic water oxidation of TiO_2_/In_2_S_3,_ has been evidenced. As shown in Figure 9b, under UV–vis light illumination, In_2_S_3_ triggered as heterostructures excited and yielded more electron–hole pairs and persuaded the water-splitting reaction at a 0 V bias. The continuous electron–hole pair parting rate with the bias potential increases the redox reaction. The photoelectrode displays a practical photon-to-electron conversion of 0.26% at 0 V vs. RHE. The visible photoexcited electrons in the conduction band (CB) of In_2_S_3_ are transported to the CB of TiO_2,_ and for an instant, the photoexcited holes from TiO_2_ are transferred to the In_2_S_3_ owing to their photo-potential to complete the reaction cycle.

As a result, water oxidation and hydrogen generation have been initiated. The solar-to-hydrogen generation from the counter electrode has been calculated as 0.33% for the TiO_2_/In_2_S_3_ photoanode.

## 5. Conclusions

In this study, we successfully developed and assembled a high-performance TiO_2_ and In_2_S_3_ layered heterostrostructure photoanode by combining the hydrothermal and CBD methods. The heterotructure nanoarrays explicitly demonstrate the superior photoelectrochemical (PEC) performance of the coupled TiO_2_ and In_2_S_3_ system, compared to the pristine TiO_2_ photoanode due to the highly efficient In_2_S_3_ heterojunction at the electrode interface. The PEC performance of the photoanodes (photocurrent evolution, electrochemical impedance, charge transport time) has been greatly enhanced by the In_2_S_3_ semiconductor particle assembly. The increase in carrier concentration (6.13 × 10^17^ cm^−3^) and the decrease in the space charge layer (10 nm) improve the charge separation efficiency of the heterostructure photoanode. The TiO_2_/In_2_S_3_ photoanode produces a maximum photocurrent of 0.28 mA cm^−2^ at −0.8 V vs. Ag/AgCl, which is a highly low onset potential for the hydrogen evolution. This enhancement results confirmed ABPE (0.33%) and STH (0.26%). The facile fabrication and the robust physicochemical evidence presented here strongly validate the TiO_2_ and In_2_S_3_ layered heterostrostructure nanoarray as a highly promising material for sustainable solar energy conversion. This successful strategy provides a clear and scalable pathway for designing next-generation photoanodes.

## Data Availability

The original contributions presented in this study are included in the article/Supplementary Material. Further inquiries can be directed to the corresponding authors.

## Supplementary Materials

The following supporting information can be downloaded at: https://www.mdpi.com/article/10.3390/nano16010044/s1. Figure S1: EDS spectrum of TiO_2_/In_2_S_3_ heterostructure. Figure S2: O1s spectrum of TiO_2_/In_2_S_3._ Figure S3: Photocurrent growth and decay time for TiO_2_ photoanode. Figure S4: Photocurrent growth and decay time for TiO_2_/In_2_S_3_ photoanode.
