# Supplementary material for: Fabrication and Photoelectrochemical Activity of In2S3 Infused TiO2 Nanorod Heterostructure Photoelectrodes for Solar Water Splitting
Source: Nanomaterials (Basel). 2025 Dec 29;16(1):44. doi: 10.3390/nano16010044 (PMC12788078; doi:10.3390/nano16010044)
Supplement: Supplementary file 1 [file nanomaterials-16-00044-s001.zip › nanomaterials-4007776-supplementary.pdf]

Electronic supplementary information

Fabrication and Photoelectrochemical Activity of In_2S_3 infused TiO_2 Nanorod Heterostructure Photoelectrodes for solar water splitting

Aravindha Raja Selvaraj [1], Kasinathan Kasirajan [2,*] and Jaehyun Hur [1,*]

[1] School of Chemical, Biological and Battery Engineering, Gachon University, Seongnam-si 13120, Gyeonggi-do, Republic of Korea; rajanano12@gmail.com
[2] Division of Advanced Materials Engineering, Kongju National University, Budaedong 275, Seobuk-gu, Cheonan-si 31080, Chungnam, Republic of Korea
* Correspondence: kasibio96@kongju.ac.kr (K.K.); jhhur@gachon.ac.kr (J.H.)

Figure captions

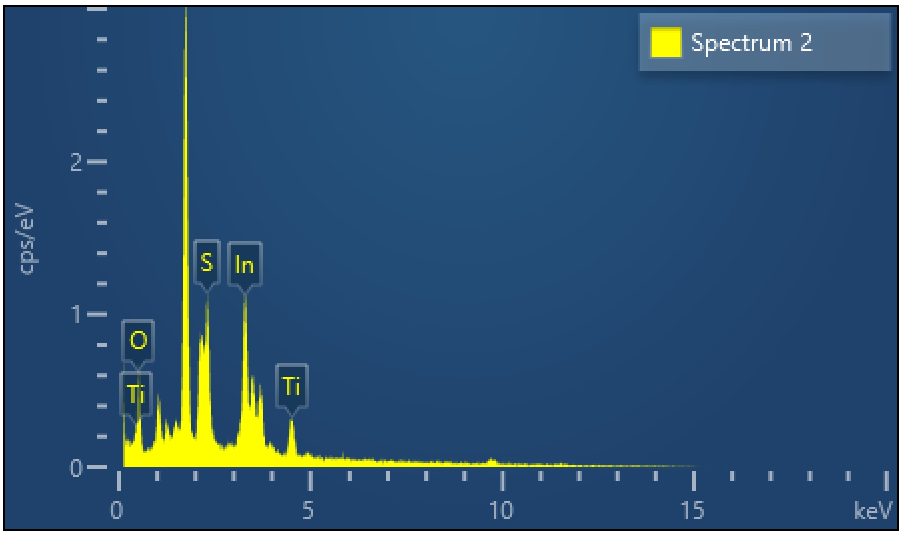

Figure S1. EDS spectrum of TiO_2/In_2S_3 heterostructure.

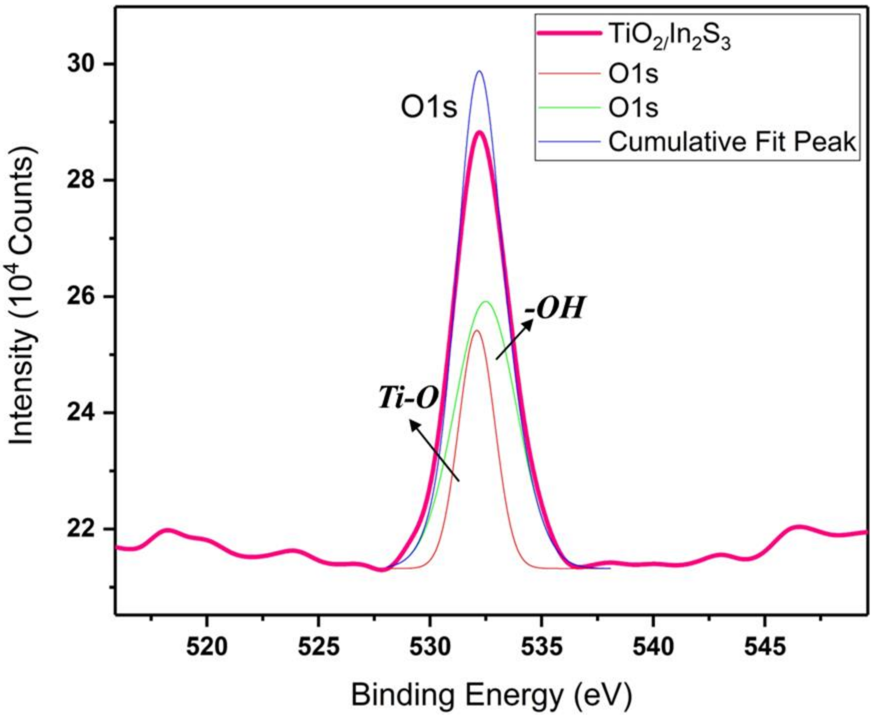

Figure S2. O1s spectrum of TiO_2/In_2S_3 photoanodes.

(b)

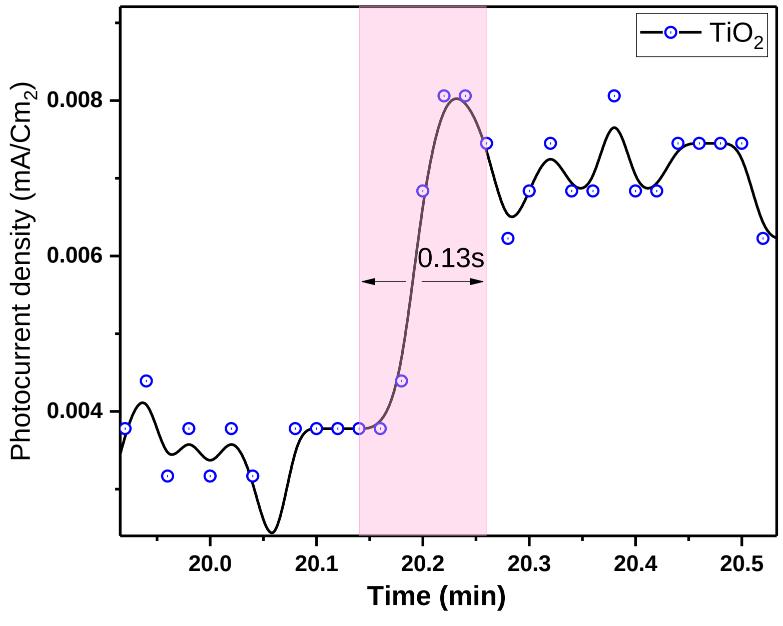

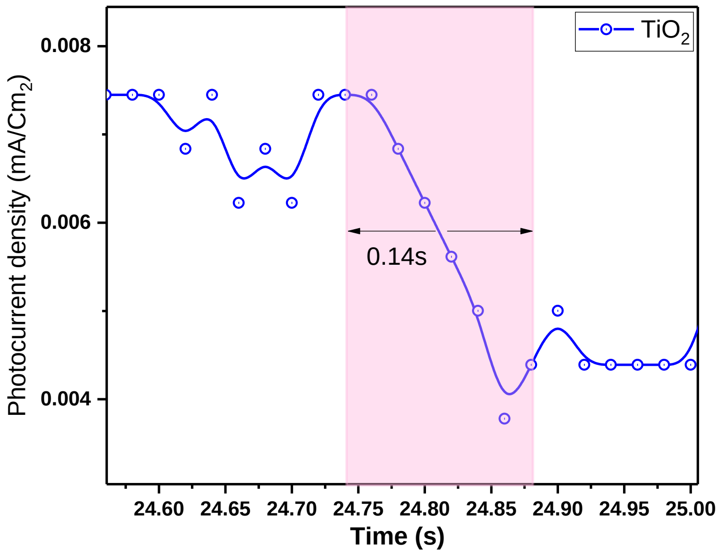

Figure S3. Photocurrent growth and decay time for TiO_2 photoanode.

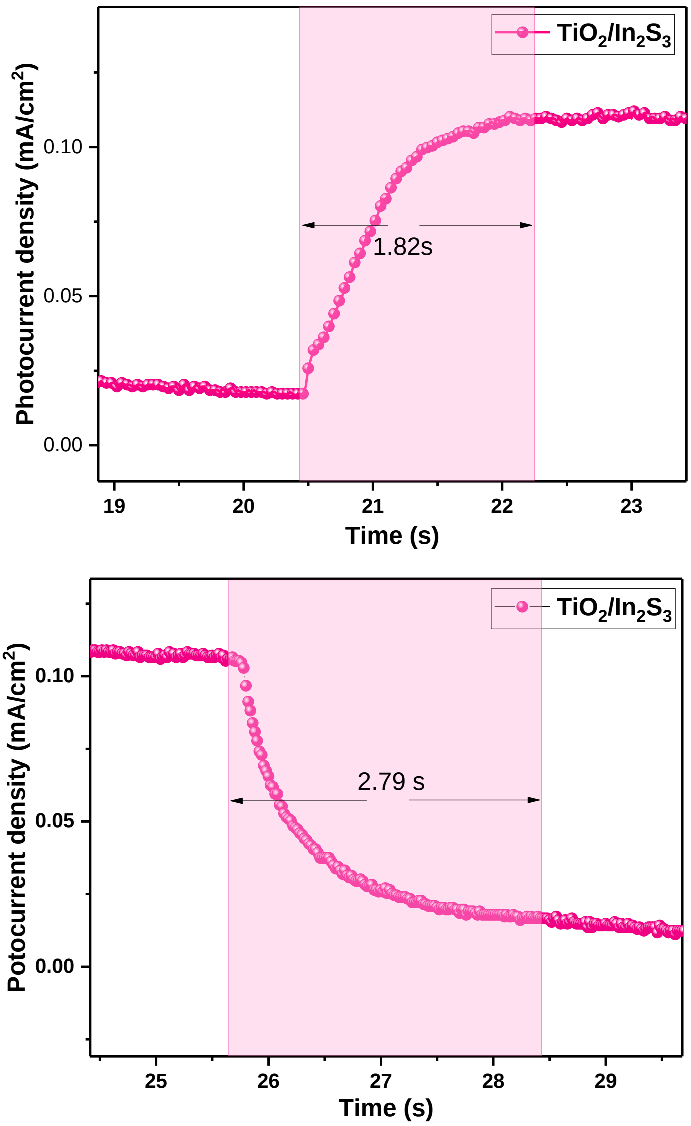

Figure S4. Photocurrent growth and decay time for TiO_2/In_2S_3 photoanode.
